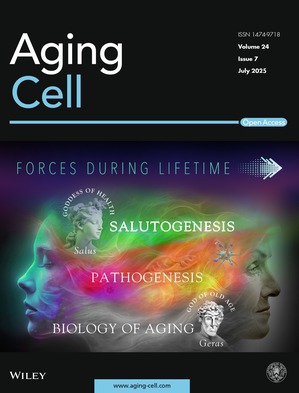# Additional Cover

**DOI:** 10.1111/acel.70172

**Published:** 2025-07-16

**Authors:** Muthu Saravanan Manoharan, Grace C. Lee, Nathan Harper, Justin A. Meunier, Marcos I. Restrepo, Fabio Jimenez, Sreenath Karekatt, Anne P. Branum, Alvaro A. Gaitan, Kian Andampour, Alisha M. Smith, Michael Mader, Michelle Noronha, Devjit Tripathy, Nu Zhang, Alvaro G. Moreira, Lavanya Pandranki, South Texas Veterans Health Care System (STVHCS) COVID‐19 Clinical team, Mohamed I. Abdalla, Sandra G. Adams, Yemi Adebayo, Joseph Agnew, Saleem Ali, Gregory Anstead, Antonio Anzueto, Marichu Balmes, Jennifer Barker, Raymond Benavides, Velma Bible, Angela Birdwell, Stacy Braddy, Stephen Bradford, Heather Briggs, Jose A. Cadena Zuluaga, Judith Marin‐Corral, Jennifer J. Dacus, Patrick J. Danaher, Scott A. DePaul, Jill Dickerson, Jollynn Doanne, Samantha Elbel, Miguel Escalante, Corina Escamilla, Valerie Escamilla, Robert Farrar, David Feldman, Debra Flores, Julianne Flynn, Delvina Ford, Joanna D. Foy, Megan Freeman, Samantha Galley, Jessica Garcia, Maritza Garza, Sherraine Gilman, Melanie Goel, Jennifer Gomez, Varun K. Goyal, Sally Grassmuck, Susan Grigsby, Joshua Hanson, Brande Harris, Audrey Haywood, Joan M. Hecht, Cecilia Hinojosa, Tony T. Ho, Teri Hopkins, Aneela N. Hussain, Ali Jabur, Pamela Jewell, Thomas B. Johnson, Austin C. Lawler, Monica Lee, Chadwick S. Lester, Stephanie M. Levine, Haidee V. Lewis, Angel Louder, Charmaine Mainor, Rachel Maldonado, Celida Martinez, Yvette Martinez, Chloe Mata, Neil McElligott, Laura Medlin, Myra Mireles, Joanna Moreno, Kathleen Morneau, Julie Muetz, Samuel B. Munro, Charlotte Murray, Anoop Nambiar, Daniel Nassery, Robert Nathanson, Kimberly Oakman, Jane O’Rorke, Cheryl Padgett, Sergi Pascual‐Guardia, Marisa Patterson, Graciela L. Perez, Rogelio Perez, Jay I. Peters, Rogelio Perez, Robert E. Phillips, Patrick B. Polk, Michael A. Pomager, Kristy J. Preston, Kevin C. Proud, Jacqueline A. Pugh, Michelle Rangel, Temple A. Ratcliffe, Renee L. Reichelderfer, Evan M. Renz, Jeanette Ross, Teresa Rudd, Maria E. Sanchez, Tammy Sanders, Kevin C. Schindler, David Schmit, Raj T. Sehgal, Claudio Solorzano, Nilam Soni, Win S. Tam, Edward J. Tovar, Sadie A. Trammell Velasquez, Anna R. Tyler, Anjuli Vasquez, Maria C. Veloso, Steven G. Venticinque, Jorge A. Villalpando, Melissa Villanueva, Lauren Villegas, Megan Walker, Andrew Wallace, Maria Wallace, Emily Wang, Stephanie Wickizer, Andreia Williamson, Andrea Yunes, Katharine H. Zentner, STVHCS COVID‐19 Vaccine team, Azaneth Arellanes, Ashley B. Banfield, Stephanie N. Bolan‐Reding, Roxanne Colazo, Katherine S. DeLeon, Norma G. Diaz, Mario A. Garza, Raed Kadhume, Hue Mang, Erwin Paleracio, Robert F. Quitta, Laura J. Ramirez, Marzieh Salehi, Cynthia J. Varela, STVHCS COVID‐19 Convalescent care team, STVHCS Center for Personalized Medicine, Andrew Carrillo, Chanda Dhami, Gaeun Jo, Krupa Jiva, Ernesto Robinson, Caitlyn A. Winter, Lauryn A. Winter, Erin Stewart, Joseph M. Yabes, Peter Melby, Cody R. Butler, Sandra Sanchez‐Reilly, Hanh D. Trinh, Clea Barnett, Luis Angel, Leopoldo N. Segal, Susannah Nicholson, Robert A. Clark, Weijing He, Jason F. Okulicz, Sunil K. Ahuja

## Abstract

Cover legend: The cover image is based on the article *The 15‐Year Survival Advantage: Immune Resilience as a Salutogenic Force in Healthy Aging* by Muthu Saravanan Manoharan et al., https://doi.org/10.1111/acel.70063.